# Prevalence of incidental premature cardiac calcifications in an HIV-infected South African population using conventional computed tomography chest radiography

**DOI:** 10.4102/sajhivmed.v22i1.1241

**Published:** 2021-05-13

**Authors:** Luize Muller, Tanusha Sewchuran, Miranda Durand

**Affiliations:** 1 Department of Radiology, Faculty of Health Sciences, University of KwaZulu-Natal, Pietermaritzburg, South Africa

**Keywords:** HIV, coronary calcification, atherosclerosis, CT, premature vascular aging, coronary plaques

## Abstract

**Background:**

International literature reported an increased prevalence of cardiovascular disease in persons living with HIV (PLWH), inferring an association with accelerated coronary atherosclerosis and plaque formation. Few local studies of HIV-related cardiac disease have confirmed this. Early identification of cardiac plaques would assist clinicians with risk stratification and implementation of treatment strategies to reduce morbidity and mortality. In resource-limited settings the use of conventional computed tomography (CT) may have a role in identifying at-risk individuals.

**Objectives:**

This hypothesis-generating study was aimed at determining the contribution of HIV to accelerated vascular aging by assessing cardiac calcifications, incidentally detected on conventional CT chest imaging, in a young HIV-positive population.

**Method:**

A retrospective quantitative analysis was performed at a tertiary hospital in KwaZulu-Natal, South Africa, over a 5-year period. Young patients (18–45 years) who underwent CT chest imaging for varied indications were included, further sub-categorised by immune status, the presence, absence and location of calcifications. Patients with unknown HIV statuses were excluded.

**Results:**

An increased probability of cardiac calcification with increasing age, independent of the HIV status, was established. No statistically significant difference could be demonstrated between the cohorts. In the pre-contrasted subcategory, a lower *P*-value suggested an ‘imminent’ statistical significance. Contrast may have obscured some calcifications. The failure to record the immune status in a large number of patients resulted in their exclusion and limited the study.

**Conclusion:**

The increased prevalence of incidentally detected cardiac calcifications in young HIV-infected individuals warrants further evaluation and cardiovascular risk stratification.

## Introduction

The life expectancy of HIV-infected individuals has dramatically increased as a result of advances in treatment, as well as in the prevention and management of opportunistic infections. Despite an improvement in immunological status and suppressed viral load, an increased risk of chronic cardiovascular disease has been documented, specifically acute coronary syndromes, cerebral vascular accidents and atherosclerosis.^1^ The exact pathophysiology is still unclear. The proposed mechanisms include insulin resistance, dyslipidaemia, endothelial dysfunction, inflammation and the direct invasion of the vessel wall by HIV.^2,3^ This increase of chronic vascular disease in an HIV-infected population will contribute to the overall morbidity and mortality, and place additional pressure on financial and operational resources. Early identification and management could result in timely intervention, the reduction of complications and improved patient outcomes, particularly in resource-poor environments where access to tertiary healthcare is limited.

Guaraldi et al.^1^ postulate that HIV causes premature vascular ageing and cardiovascular disease. An indicator of this is the ‘unexpected’ presence of atherosclerotic plaque in youth and young adults when usually only encountered in those aged ≥ 50 years. Radiographic imaging and evaluation of the characteristics of the plaque assist with determining the overall severity.^1^ Many international studies have demonstrated an increased incidence of coronary plaque in persons living with HIV (PLWH), mostly uncalcified and often associated with poorer clinical outcomes.^1,3,4,5,6,7,8^ Little of this data is available from Africa, despite sub-Saharan Africa being severely affected by HIV. The finding of an increased prevalence of premature cardiac calcifications in South Africans (SA) living with HIV will inform the need for early detection, intervention and prevention to reduce long-term cardiovascular-related morbidity and mortality.

Valvular calcifications have also been linked to vascular ageing and accelerated atherosclerosis.^9,10,11^ Lange et al.^9^ determined that both mitral annular and coronary artery calcifications were amplified in PLWH. The authors surmised that the concomitant presence of mitral and coronary artery calcifications was associated with morbidity and mortality.^9^ A more recent analysis from the Multicentre AIDS cohort study^11^ found that combined mitral and aortic valvular calcifications were more prevalent in their HIV-infected cohort.

**FIGURE 1:**
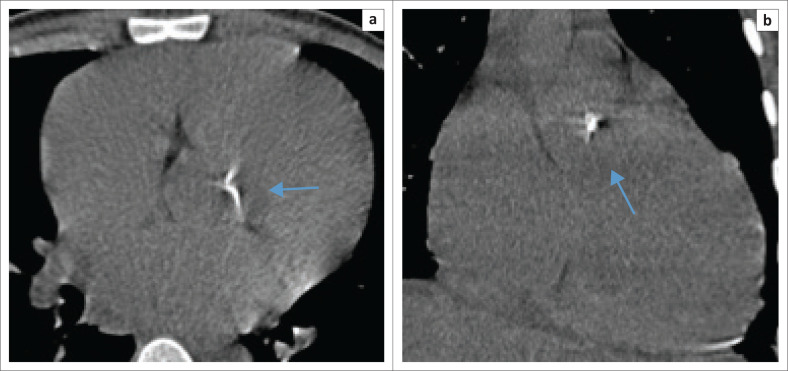
Axial (a) and coronal (b) precontrasted images through the mediastinum depicting dense calcium deposition on the aortic valve as indicated by the arrows.

**FIGURE 2:**
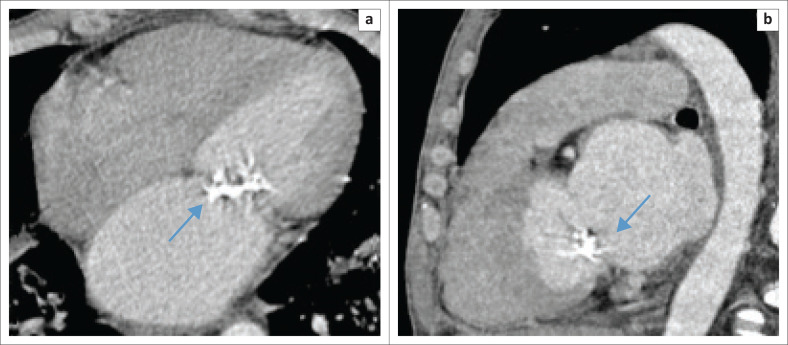
Axial (a) and sagittal (b) contrasted images of the heart demonstrating coarse mitral valve celcifications as indicated by the arrows.

Of particular note was the high incidence of uncalcified plaques in the coronary arteries of patients who presented with aortic valve (AV) calcifications (Figures 1 and 2). This finding suggests that despite conventional computed tomography (CT) not being able to reveal uncalcified coronary plaque, the presence of AV calcifications suggests that additional investigation is warranted.^11^

Both coronary artery plaque and valvular calcification, which are considered objective indicators of atherosclerosis, can be evaluated with imaging. Previous studies examined the relationship between atherosclerotic plaque and HIV with the use of electrocardiogram (ECG)-gated cardiac CT and coronary angiograms. The plaque burden was quantified using the Agatston calcium score.^2,4,6,12^ This method requires dedicated and specialised hardware and software not readily available in all hospitals, particularly in resource-constrained centres. In most developing countries, where the disease burden is often the highest, conventional spiral CT is usually a more accessible and affordable alternative.

A study conducted by Chandra et al. made use of the Weston score as an alternative for calcium scoring.^13^ The Weston score does not rely on specialist equipment or contrast. Coronary plaques are identified on non-contrasted images obtained by conventional CT, and a scoring system is used to classify the type of calcification. While the Weston score is more subjective, because it relies on the interpreters’ visual identification and requires good quality unenhanced imaging, it can be used as a possible alternative to detect calcifications with similar outcomes.^13^

South Africa has a high prevalence of HIV infection principally in adolescents and young adults, aged 15–49 years. In view of the improved life expectancy of SA-LWH, many of whom were infected at an early age, we investigated the prevalence of premature cardiac calcifications (coronary and valvular) incidentally detected in groups of young SA-LWH and using a control group of HIV-negative individuals of similar age for comparison. By evaluating patients younger than 50 years, the traditional risk factors associated with vascular ageing are less likely to be active. We further recommend and support the use of conventional spiral CT chest (contrasted or non-contrasted) as an alternative to ECG-gated CT in a resource-constrained setting, to screen for incidental cardiac calcifications.

## Research methods and design

Our study was based on the hypothesis that HIV prematurely accelerates cardiac ageing. We performed a retrospective quantitative analysis of all patients between the ages of 18 and 45 years who underwent a chest CT scan during a 5-year period (01 January 2014 – 31 December 2018) at a tertiary hospital in KwaZulu-Natal. These scans were not dedicated cardiac CTs, simply because in resource-limited settings such as ours, we do not routinely perform ECG-gated chest imaging. Indications for CT chest were therefore varied, included both medical and surgical and were not specifically cardiac in nature. Patients seen during normal working hours and after hours were included. No specific patient preparation, such as the use of a beta blocker, was administered to alter the cardiac rate or reduce motion artefacts. Patients were scanned on a Somatom Sensation 64-slice cardiac CT scanner using standardised imaging parameters. Images were accessed from the picture and archiving communication system (PACS). The CT imaging protocol included pre-contrast only or both pre- and post-contrast, plus a routine 100-mL bolus of Omnipaque 300 contrast at a flow rate of 3 mL/s. The date of the scan, age, gender and HIV status were collected retrospectively from the CT request forms. In the event that the patient’s HIV status was not provided on the request form, laboratory confirmation was sought from the National Health Laboratory Service (NHLS) database.

Two board-certified radiologists, with a specialist interest in cardiothoracic imaging and with 5 and 10 years of experience, reviewed the preselected and anonymised studies and recorded, with the aid of a data collection tool, the presence or absence of cardiac calcifications in patients referred for imaging (Appendix 1). Where interobserver variability occurred, the images were reviewed and consensus was sought. The exact location of the calcifications (coronary and valvular) and the imaging technique (pre- or post-contrast chest CT) were available for evaluation. The evaluated vascular sites included the left marginal artery (LMA), left anterior descending artery (LAD), right coronary artery (RCA), and left coronary artery (LCA). The valvular sites included the mitral valves (MV) and AV. The radiologists were blinded to the patient’s HIV status. Patients were excluded from the study if their HIV status was unknown or if the CT scan was considered non-diagnostic, for example, if a significant artefact obscured the anatomical regions of interest.

Descriptive statistics were used to differentiate the demographic and clinical characteristics of the patients, sub-categorised into HIV-positive and HIV-negative. These included specific age categories and gender.

Furthermore, a statistical analysis was performed on the pre-contrasted studies only in order to determine the estimated probability of developing cardiac calcifications. The presence of cardiac calcifications between the two groups was compared using a chi-square test. The effects of age and gender were adjusted by using a logistic regression model. A *P*-value of 0.05 or less was regarded as statistically significant, with a confidence interval of 95%.

### Ethical considerations

Ethical approval was obtained from the Research Ethics Committee of the Faculty of Health Sciences, the Department of Health and the medical manager of the hospital where the research was conducted.

All images were captured using Grey’s Radiology PACS with the permission of the medical manager, ethics committee and head of the Department of Radiology.

## Results

**FIGURE 3:**
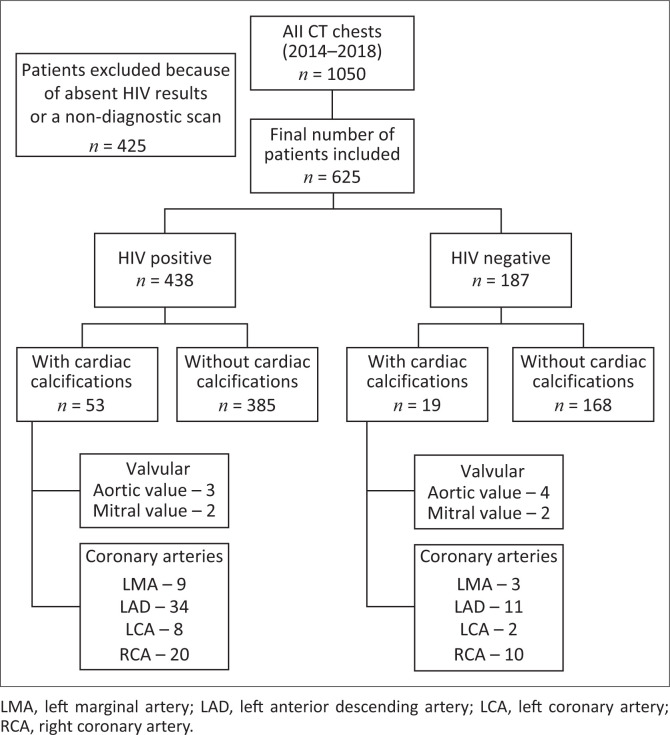
Diagrammatic representation of the results.

A total of 1050 patients underwent CT of the chest during the 5-year period (01 January 2014 – 31 December 2018). Four hundred and twenty-five patients were excluded because their immune status was not documented or because of non-diagnostic scans with poor image quality. A total of 625 patients with a documented HIV status were included in the final study sample. Of the 438 patients who were HIV positive, 53 (12.1%) patients demonstrated cardiac calcifications. In the HIV-negative sample, consisting of 187 patients, 19 (10.2%) had documented cardiac calcifications (Figure 3). The difference in the percentage of HIV-positive and HIV-negative participants with calcifications was not statistically significant (*p* = 0.487). The discrepancy in the overall size of the respective populations limits statistical accuracy but suggests the need for further prospective analysis.

An increased probability of calcification with increasing age was noted, independent of HIV status (*p* = 0.00006). In any multiple regression model where the independent variables are mutually uncorrelated, the estimated coefficients associated with the independent variables relate to those variables only. Because calcification was not significantly correlated to HIV status in our limited study, we can claim that any conclusion referring to whether a patient shows calcification is not affected by that patient’s HIV status.

No statistically significant difference was noted between gender, HIV status and the probability of developing calcification (logistic regression, *p* = 0.281 and *p* = 0.952) (Table 1). The median age of the patients in the HIV-negative group was 31 years and in the HIV-positive group, it was 36 years.

**TABLE 1:** Estimated probability of calcification as a function of age, gender and HIV status.

Calcifications	*P*-value		
	Age	Gender	HIV
Total calcifications	0.00006	0.281	0.952
**Valvular**			
Aortic valve	0.64030	0.84154	0.11353
Mitral valve	0.6333	1.0	0.58386
**Coronary arteries**			
LMA	0.0539	0.08357	0.76534
LAD	0.00230	0.26469	0.687166
LCA	0.048	0.18664	0.86677
RCA	0.00598	0.22721	0.44985
Calcification seen on pre-contrasted images only	0.04002	0.40333	0.077129

LMA, left marginal artery; LAD, left anterior descending artery; LCA, left coronary artery; RCA, right coronary artery.

**FIGURE 4:**
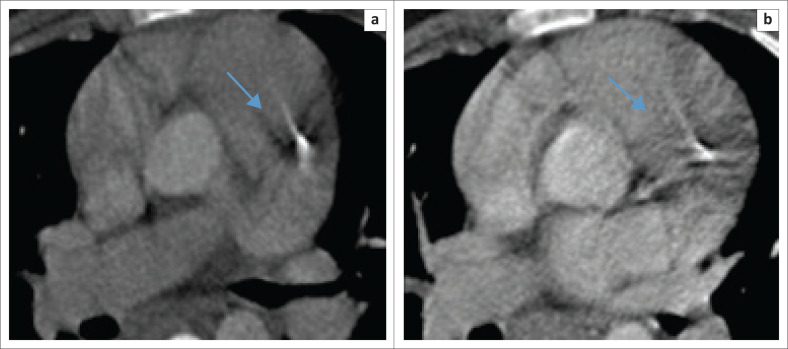
Post-contrasted axial images (a & b) at different levels showing calcifications along the left anterior descending coronary artery as indicated by the arrows.

Regarding the anatomical location of the calcifications, no statistical significance was noted between the HIV-positive and HIV-negative groups, particularly in the LAD coronary artery, where the majority of calcifications were detected, likely because of its anatomical visibility (*p* = 0.40502) (Figure 4).

**FIGURE 5:**
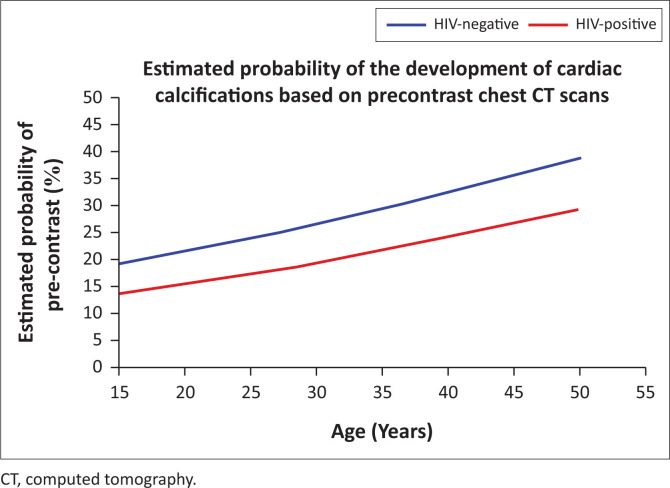
Demonstrates an increased probability to develop cardiac calcifications with an increase in age.

In the AV, 2.1% of the HIV-negative sample compared to 0.7% of the HIV-positive sample demonstrated calcifications, with a difference of 1.4%, which was not statistically significant (*P* = 0.13699) (Figure 5, Table 2).

**TABLE 2:** Comparison of the probability to develop cardiac calcification between HIV positive and HIV negative cohort groups.

Calcifications	Percentage HIV+	Percentage HIV-	*P*-value
Total calcification	12.1	10.2	0.48663
**Valvular**			
Aortic valve	0.7	2.1	0.113699
Mitral valve	0.5	1.1	0.378941
**Coronary arteries**			
LMA	2.1	1.6	0.707047
LAD	7.8	5.9	0.405022
LCA	1.8	1.1	0.489809
RCA	5.3	4.6	0.675619

LMA, left marginal artery; LAD, left anterior descending artery; LCA, left coronary artery; RCA, right coronary artery.

In total, 619 patients had pre-contrasted chest imaging. Within this subdivision, 95 patients with visible cardiac calcifications were HIV-positive, and 51 were HIV-negative, a percentage difference that was not statistically significant (*P* = 0.1409).

The lower *P*-value provides some weak evidence that increasing age also increased the probability of an affirmative when assessing the pre-contrasted images only. This is suggestive but inconclusive evidence that HIV status might have an effect. The dense contrast may obscure soft calcifications and implies that further investigation is warranted.

**FIGURE 6:**
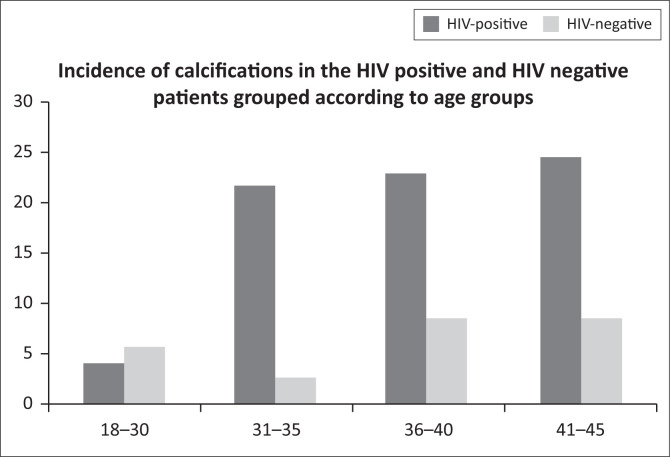
Demonstrates the numerical variance, in percentage, in the incidence of calcifications in the different age categories according to immune status.

A noticeable numerical variance was seen in the incidence of cardiac calcifications between the HIV-positive and HIV-negative cohorts, when evaluating the different age categories, specifically seen in the age group of 31–35 years (Figure 6).

## Discussion

Atherosclerotic plaques are a strong indicator of vascular ageing, a finding commonly seen in individuals over the age of 50 years. However, it has been well documented internationally that the long-term effects of HIV in PLWH, who now have improved life expectancy, include coronary ageing at an accelerated pace.^1^ The burden of HIV and AIDS in Southern Africa has an earlier influence on the population, with the likelihood of an increase in premature vascular ageing.

In an attempt to rule out the effects of normal vascular ageing and other comorbidities associated with advanced age, a younger population was chosen, with a median age of 31 in the HIV-negative and 36 in the HIV-positive patients.

Fitch et al. demonstrated an association between accelerated cardiovascular ageing and the immune status of the HIV-infected patient.^5^ The presence of cardiac calcifications in a much younger population (< 50 years) signifies premature vascular ageing in HIV-infected individuals.

Even though the percentage of participants with calcifications was not significantly different between the groups, the mere presence of calcifications in the younger age group is concerning for the development of premature atherosclerotic changes.

By assessing the probability of developing calcifications in the two study groups on the pre-contrasted images only, there was weak but inconclusive evidence that HIV might have an effect. This suggests that by eliminating the effect of contrast artefacts, the visibility of calcifications would be increased, yielding more conclusive data.

Furthermore, Rezaeian et al. proved a higher incidence of non-calcified atheromatous plaques in an HIV population, a finding that cannot be demonstrated with conventional CT.^11^ By looking at calcifications only as a measure of vascular ageing, we excluded patients with non-calcified atheromatous plaques by default, making it difficult to assess the true extent of vascular ageing in our study sample.

Using ECG-gated cardiac CT would have improved the detection of non-calcified plaques and would also have reduced motion artefacts, which could further improve identification.

In combination, the above-mentioned findings do not exclude a link between HIV status and the development of premature cardiac calcifications. The possibility remains and warrants further investigation, as does the prevalence of uncalcified plaque in this population – an entity that has been internationally recognised with growing concern.

Our study further incidentally emphasises the known association of increasing age with the development of cardiac calcifications and underscores that their presence can be used as an objective indicator of vascular ageing.

## Limitations

Our study aimed to assess the effect of HIV on premature cardiac ageing, observed as incidentally detected valvular and coronary calcifications in young patients. The retrospective review of CT chest scans performed for other indications in patients younger than 45 years of age, in whom comorbidities are thought less likely, limited diagnostic accuracy. Furthermore, the lack of preparation for imaging, such as the use of beta blockers, and the relatively small sample size are further considerations. While our sample size is small, the positive findings in the HIV-infected group are concerning and warrant further investigation.

We unfortunately did not take into account the viral load, CD4 status, time since diagnosis or the use and type of antiretroviral drugs in our patient population. This is an important limiting factor as certain treatment regimens have been found to contribute to vascular ageing.^3^ Furthermore, the time since HIV diagnosis, as some patients may have seroconverted shortly before imaging, may result in less time to develop calcified plaques or even non-calcified atheromatous plaques.

Lastly, the unavailability of immune status proved a significant limiting factor. A large number of patients in our initial sample size had no documented HIV status on the referral letter, with no serological test results available on the NHLS system. Because of this, a significant number of patients were excluded from the study, resulting in a mismatch in numbers between the cohort groups.

## Conclusion

Our hypothesis of increased cardiac calcifications in young HIV-infected individuals, incidentally detected on CT chest radiography, shows distinct promise and definitely warrants further imaging, best with a prospective study detailing cardiovascular risk stratification in this select population, which will positively impact patient outcomes.

## Appendix 1

**Table:**

Nr	Patient GR number	Age	Gender	Calcifications	Valvular		Coronary arteries			
					Aortic	Mitral	LMA	LAD	LCA	RCA
1.	-	-	-	-	-	-	-	-	-	-
2.	-	-	-	-	-	-	-	-	-	-
3.	-	-	-	-	-	-	-	-	-	-
4.	-	-	-	-	-	-	-	-	-	-
5.	-	-	-	-	-	-	-	-	-	-

LMA, left marginal artery; LAD, left anterior descending artery; LCA, left coronary artery; RCA, right coronary artery.
